# Bibliography

**Published:** 1899-12

**Authors:** 


### CORRECTION.

In the obituary article on Dr. Bonwill, in the November number, two letters dropped out of the word *prostatitis*, to the annoyance of the editor. The intelligent reader no doubt supplied the omission.

### Bibliography.

An Epitome of the History of Medicine. By Roswell Park, A.M., M.D., Professor of Surgery in the Medical Department of the University of Buffalo, etc. Based upon a course of lectures delivered in the University of Buffalo. Second Edition. Illustrated with Portraits and other Engravings. Pages 370. The F. A. Davis Co., Publishers, Philadelphia.

This valuable history of medicine was fully reviewed in this journal upon the appearance of the first edition, and that the second is called for within a year is confirmatory of the favorable opinion then given and of the interest with which it has been received by the medical profession.

This edition shows but few changes made. With the exception of the correction of some "inaccuracies" in the first edition, to which the attention of the author had been called by reviewers, and the addition of a supplementary chapter on "Iatrotheurgic Symbolism," there is but little added. This latter addition is, however, an exceedingly interesting one, although the propriety may be questioned of introducing it in a work on the history of medicine. While it may be considered as straining a point to regard the phallic emblems as in close relationship to medicine, the historical student will not deem this important in view of the value of the effort of the author to establish the full value of the symbols that have come down to us from so-called pagan sources.

While dentistry has reason to be grateful for the chapter upon this subject, the reviewer cannot avoid the regret that this had not been made more complete, but as far as it goes due credit is given to the work of this profession, and the author deserves unstinted praise for his willingness to consider the record made in dentistry as not unworthy a place in his history of medicine.

The history of dentistry cannot be written apart from that of medicine, hence dentists, in order to rank as men of culture must familiarize themselves with its history, from which they can alone date the origin of their profession. Dentistry has not yet reached the standard set up by the Greek anatomist Erasistratus when he deposited in the temple of the Delphian Apollo the leaden forceps (Odontogogue) to indicate that a tooth should not be extracted until it could be removed by that character of instrument. That act, so far-reaching in principle, should be well studied, and perhaps in the twentieth century we may discover that the advice given two thousand three hundred years ago has become a fact in practice.

The work has been prepared by one having the faculty of condensation without sacrificing the salient points of medical history, while at the same time the author has been able to give his readers a work full of interest from the first page to the last.

THE HYGIENE OF THE MOUTH: A GUIDE TO THE PREVENTION AND CONTROL OF DENTAL DISEASES. By R. Denison Pedley, F.R.C.S. (Edin.), L.D.S. (Eng.), Dental Surgeon to the Evelina Hospital for Sick Children, Southwark, London. Numerous Illustrations. J. P. Legg & Co., London, and S. S. White Dental Manufacturing Company, Philadelphia.

This book of ninety-three pages is an effort to show "that dental diseases are widely prevalent, that they are the sources of much misery, and have serious effects upon the general health from early life to mature age." At the first glance this explanatory paragraph from the author's preface would seem to indicate that his book must be a series of recognized facts and hardly worth the effort required to bring them to the attention of dental readers. While the latter are supposed to be familiar with the teachings of this book, they are by no means over-intelligent regarding much condensed within its pages, nor are they sufficiently careful to instruct their patients in the means best adapted to keep the mouth in a hygienic condition. The author, therefore, has performed a real service to dentistry in marshalling his facts, and could the book be published in cheap paper covers, so that dentists generally could have it, or, what would be better, have abstracts made for general circulation, it would have a value not attainable in its present form. The ignorance of the masses in regard to hygiene of the mouth is profound. To educate in this direction is one of the greatest necessities of this age; but this fact is not appreciated to its full importance by dentists, and certainly not to any extent by the laity.

The author dwells, very properly and at length, on the care of the deciduous teeth. A very excellent and effective illustration is given on page 7 showing the action of the tooth-brush over the convex surfaces of the teeth. It tells its own story better than a page of descriptive text.

The author seems to give undue prominence to caries as a factor in producing epilepsy, chorea, etc. While it is unquestionably true that caries may produce these reflex disturbances through exposure of pulps, it is doubtful whether in all the cases reported the nervous conditions had their origin from this cause. One case is mentioned on page 23 in which the patient was affected with epilepsy at nine years of age. Six carious teeth were removed, with entire relief. The explanation the reviewer would give of this case would be, that the second molars, at this age, were impinging upon the inferior dental nerve, and that these, and not caries, were the cause of the nervous disturbance. The removal of the first permanent molars allowed the second molars to come forward more rapidly, and the irritation ceased.

The effect of the products of putrefaction are lucidly set forth under the proper heading, accompanied with cases and statistics sufficient to startle the ordinarily unobservant mind.

If this book could be read chapter by chapter in our public schools, taking the place of some other reading matter, it would be a cause of less contention and more of a blessing to the pupils, as well as of value in insuring a greater degree of health to future generations.

The ignorance of the general public in regard to the care of the teeth is simply appalling, and it is time that organized dentistry should make an effort, through cheap tracts, to disseminate knowledge in this direction. Money is always wisely expended when it adds to the health and comfort of the masses. This work of R. Denison Pedley points the way to a better understanding of the hygiene of the mouth, and is worthy of careful reading by all classes of the community.

